# Analyzing the effects of climate risk on discouraged borrowers: Deciphering the contradictory forces

**DOI:** 10.1111/risa.15071

**Published:** 2024-07-15

**Authors:** Dimitris Anastasiou, Antonis Ballis, Christos Kallandranis, Faten Lakhal

**Affiliations:** ^1^ Department of Business Administration Athens University of Economics and Business Athens Greece; ^2^ Aston Business School Aston University Birmingham UK; ^3^ Department of Accounting and Finance University of West Attica Athens Greece; ^4^ Léonard de Vinci Pôle Universitaire, Research Center Paris France

**Keywords:** climate risk, credit markets, discouragement, eurozone SMEs, SAFE

## Abstract

We examine the impact of climate risk on discouraged borrowers among small and medium‐sized enterprises (SMEs) in the eurozone, using a unique European Central Bank dataset focusing on the demand side of credit markets. We argue that two opposing channels may exist in this relationship: Either climate risk has a negative effect stemming from increased demand for sustainable or climate‐resilient projects that enhance creditworthiness, or climate risk has a positive effect arising from heightened climate uncertainty and risk aversion, leading to credit self‐rationing among SMEs. Our findings reveal that heightened climate risk prompts SMEs to self‐ration credit, leading to higher probabilities of discouraged borrowers. Our research deepens the understanding of the impact of climate risk on credit‐related decisions, stressing the need for proactive measures to integrate climate risk assessments into regulatory frameworks and lending practices. The findings underscore the vulnerability of SMEs to climate risk, emphasizing emphasizing the importance of tailored support mechanisms for economic resilience.

## 1 INTRODUCTION

Since the implementation of the Paris Agreement in 2015, there has been rising interest in the climate change agenda. Unfavorable weather conditions are being clearly carefully recorded, given that droughts, extreme temperatures, floods, and storms, among others, pose a huge threat to humanity (Eckstein et al., 2019; Kling et al., 2021) and lead to profound economic and human losses (Zhang & Shunsuke Managi, 2020). Indeed, over the period 1999–2018, there were more than 10,000 extreme weather events worldwide, resulting in more than 475,000 deaths and direct losses of approximately US$2.56 trillion (Eckstein et al., 2019). According to the International Disaster Database, a total of 432 disastrous events were recorded worldwide in 2021, which caused 10,492 deaths and approximately US$252.1 billion in economic losses. Furthermore, research suggests that a temperature change of 1.5°C could result in a 70%–83% increase in human casualties from flooding and a 160%–240% increase in flood damage (Dottori et al., 2018). Hence, the negative effects of climate change on our planet can no longer be ignored (Bartram et al., 2022; Calvet et al., 2022). The increasing global focus on mitigating the negative consequences of climate change has led governments to emphasize the importance of addressing climate risk by reducing greenhouse gas emissions and prioritizing adaptative measures, in line with the Paris Agreement. Recently, the Pörtner and Belling (2022) and The Sustainable Development Goals Report (2023) urged civil society and companies to contribute to climate change resilience.

The anthropogenic issue of climate change is highly complex and over‐changing making past data ineffective in reflecting current conditions. The general sense of fear about climate change has triggered a massive spike in uncertainty in people's lives, and its spread has caused enormous negative economic consequences, creating an additional threat to the resilience of global economic activity (Botzen et al., 2019; Cavallo et al., 2013; Kahn et al., 2021; Kling et al., 2021). Examining the evidence of the negative effects of uncertainty shocks on economic activity has been at the forefront of empirical research (Baker et al., 2016; Bordo et al., 2016; Shin & Zhong, 2020). Such uncertainty affects the decisions of individuals, financial intermediaries, governments, and firms at varying levels. However, as Knight (1921) wrote, “a known risk is easily converted into an effective certainty, while true uncertainty is not susceptible to measurement.” In the framework of climate change, this distinction might be overblown.

Given the myriad uncertainties surrounding climate change, it is critical to explore the association between climate risks and both micro‐ and macroeconomic conditions. Chen et al. (2022) found that climate risks have significant impacts on capital inflows in developing countries. Battiston et al. (2021) suggested that climate risk has been recognized as a new source of instability in the financial system. Companies are becoming more cautious about climate change because it exposes them to several business risks, ranging from supply chain disruptions and increased operational costs to reduced demand for goods and services (Campiglio et al., 2018; Lamperti et al., 2019). Researchers have recently explored several adverse effects of climate risk on companies, including disruptions in operations and damage to corporate assets (Hugon & Law, 2019), financial performance (Addoum et al., 2020; Pankratz et al., 2023), capital structure (Ginglinger & Moreau, 2023), cash holding (Javadi & Masum, 2021; Yu et al., 2022), the cost of equity capital (Huynh et al., 2020), earnings management (Ding et al., 2021), tax avoidance (Ni et al., 2022), and the cost of debt (Delis et al., 2024).

Although scholars are increasingly interested in the relationship between climate risk exposure and economic activity, the influence of climate risk on credit markets, which are not isolated from these shocks, appears to be comparatively unexplored. Although natural factors drive climate conditions and are largely exogenous to firms, the overall negative sentiment due to the uncertainty surrounding climate risks can reduce credit demand as fewer firms being unsure about their future prospects and financing needs and request fewer loans. According to the real options theory, firms typically become more cautious during periods of high uncertainty (An et al., 2016; Bloom, 2009; Bloom et al., 2007; He et al., 2022). As a result, they postpone making significant decisions about capital budgeting and financing. This can ultimately lead to a decline in firms’ financial performance and investment returns (Engle et al., 2020; Matsumura et al., 2014). In this respect, we contribute to the literature on uncertainty and credit markets by encapsulating the climate risk factor and its effect on agents’ credit‐related decisions. Although the prominent role of supply‐side problems in loan provision is acknowledged (Delli Gatti et al., 2003; Mishkin, 2011), our article exploits cross‐country microlevel data to highlight the aspects of climate risk that best explain falls in credit demand.

The financing decisions of small and medium‐sized enterprises (SMEs) are the perfect context for our investigation. Indeed, SMEs exhibit a high reliance on bank credit as their limited size prevents them from accessing the capital markets (Banerjee & Duflo, 2014; Beck & Demirguc‐Kunt, 2006; Berger & Frame, 2007; Berger & Udell, 1998; Drakos & Kallandranis, 2005; Kallandranis et al., 2023). In comparison to large enterprises, SMEs are less flexible in the face of unexpected shocks and are less capable of hedging against uncertainty as they are subject to more financial constraints during tight periods (Amoroso et al., 2017; Calabrese et al., 2022; Ghosal & Ye, 2015).

Although there is adequate evidence on the relationship between climate risk and corporate financial distress, there is insufficient evidence on the relationship through which climate change shocks are propagated among a shadow category of loan applicants, those identified as discouraged borrowers. According to Kon and Storey (2003), discouraged bank borrowers refer to enterprises that opt not to pursue a bank loan despite their need for it, mostly driven by their aversion to rejection. The occurrence of discouragement holds considerable importance in credit markets, and the prevalence of discouraged bank borrowers is twice as high as that of rejected borrowers (Cowling & Sclip, 2022; Freel et al., 2012; Kallandranis et al., 2023). The level of discouragement among SMEs tends to rise during recessions or times of uncertainty because banks tend to cut their lending (Ivashina & Scharfstein, 2010). Discouraged borrowers pose significant challenges to policymakers and macroeconomic and financial institutions, hindering their ability to invest and benefit from financial opportunities. The potential ramifications of this phenomenon on economic growth and development are predominantly adverse (Kallandranis & Drakos, 2021; Kallandranis et al., 2023). Therefore, it is crucial for policymakers and financial institutions to develop effective strategies to improve loan accessibility, which will contribute to economic growth and development (Vermoesen et al., 2013). Previous studies have investigated various factors related to borrowing discouragement, such as the Global Financial Crisis (Cowling et al., 2016), CEO gender diversity (Forrester & Neville, 2021), corporate innovation (Brown et al., 2022), firm productivity (Jaulín‐Méndez, 2022), credit constraints (Ferrando & Mulier, 2022), and economic sentiment (Anastasiou et al., 2022). However, the impact of climate risk as an additional risk factor on discouraged borrowers remains unexplored.

The lens we look through in this article is that of the debt market for SMEs. We analyze 27 waves of cross‐sectional data on eurozone SMEs from 2009 to 2022. Our primary research question is whether climate risk leads managers to self‐ration credit. We report that when SME borrowers feel uncomfortable in environments characterized by higher climate risk, they are deeply concerned about being able to repay their creditors, leading to an intensified fear of rejection.

Our results contribute to the broad and growing literature on SME financing decisions and borrower discouragement, skipping the overexamined credit supply side and focusing exclusively on the credit demand side and cross‐country evidence. Using a rich sample of a unique, confidential, survey‐based dataset of the European Central Bank (ECB) focusing on a category of SMEs that has been neglected thus far, we develop an econometric model for discouragement that accounts for firm‐specific factors to capture the objective component of firms’ assessed chance of loan application success. We augment the econometric model using the climate risk component and test whether it has any explanatory power in relation to discouragement. To the best of our knowledge, this is the first article to explore how climate risk shapes the discouragement of firms to borrow. We present novel findings demonstrating that climate risk has major consequences for credit‐related decisions and that a new mechanism must be implemented in order to protect economies from climate shocks.

The rest of the article is structured as follows. Section 2 briefly discusses the theoretical background and related empirical studies in the literature. Section 3 describes the data and methodology. Section 4 presents our empirical findings, and Section 5 concludes the article.

## 2 LITERATURE REVIEW AND THEORETICAL BACKGROUND

### 2.1 Literature review

Under a Modigliani and Miller (1958) framework, any friction such as climate risk should play no role in firms’ financial decisions (Ginglinger & Moreau, 2023). However, the collapse of the Modigliani and Miller theorem in the presence of either asymmetric information or agency costs (Jensen & Meckling, 1976) led to the conclusion that such a phenomenon in debt financing may increase the cost of new debt or even restrict firms from borrowing due to credit rationing (Greenwald et al., 1984; Stiglitz & Weiss, 1981). The origins of contemporary credit market analysis, as viewed through the lens of imperfect information theory, may be traced back to the work of Jaffee and Russell (1976). In their study, these authors illustrate how undisclosed variations in the quality of borrowers can lead to the imposition of limitations on loan availability. The crucial unobserved factor pertains to the ex‐ante risk associated with loan applications from prospective borrowers and the subsequent interest rate determined by banks. These factors might give rise to either adverse selection or incentive/moral hazard effects. Such a situation can cause profitable investment projects to be postponed or even cancelled, fueling the subsequent negative effects on economic activity.

It is clear that the existing body of financial research has predominantly concentrated on arguments that attempt to shed light on the financing challenges SMEs face by attributing them simply to a weakness in accessing credit supply. However, this literature has largely overlooked the factors that restrict the demand for resources in these firms. Drawing on the general theories of credit rationing, Kon and Storey (2003) presented a concept that focuses on interpreting the existence of discouraged borrowers. In this context, the discouraged borrower model posits that a firm, irrespective of its quality, will only seek a loan if the return on an investment project is above the effective borrowing cost (Cowling et al., 2016). Indeed, a considerable proportion of SMEs opt against requesting a bank loan, despite their financial need for one, out of concern regarding potential rejection (Cavalluzzo & Wolken, 2005; Chakravarty & Xiang, 2013; Cole & Sokolyk, 2016; Freel et al., 2012; Kallandranis et al., 2023; Rostamkalaei et al., 2020).

Going beyond the typical financial aspect of credit self‐rationing, this study explores a related and equally interesting dimension of this phenomenon: climate risk. We aim to determine how climate risk, as an additional factor of uncertainty, affects SME decision‐making and, in particular, their financing decisions when applying for loans. The discouragement of borrowers, given many investors’ shorter‐term perspectives, is often assessed in ways that fail to encompass exogenously driven climate threats, which are intricate and unclear and often lack precedent. The analysis exposes the so far underappreciated climate change factor in SMEs’ borrowing decisions and its subsequent effects on economies.

The macroeconomic literature provides broad evidence on the significant impacts of climate risk on different aspects of economies. Battiston et al. (2021) argued that climate change may lead to financial system instability as it is considered to be a new source of risk. Jones and Olken (2010) showed that climate change has led to a decrease in the growth rate of countries’ exports. According to Fankhauser and Tol (2005), the adverse effects of climate change are harmful for economic growth and future welfare. Burke et al. (2015) also provided evidence of the intricate link between rising temperatures and global economic productivity and activities and emphasize the urgent need for effective climate change mitigation.

A growing body of literature examines firm‐level evidence on the impacts of climate risk. For instance, Ozkan et al. (2023) showed that firms exposed to greater climate risk experience a significant decrease in firm performance and that corporate social responsibility mitigates the decrease in performance related to climate change. Similarly, Pankratz et al. (2023) documented that increased exposure to extreme weather events reduces firms’ sales and operating income. Addoum et al. (2023) demonstrated that temperature shocks negatively impact firms’ earnings and stock market returns, respectively. Moreover, the various uncertainties caused by environmental changes lead to high stock market volatility (Gros et al., 2016). The adverse effects of climate change can also result in financial challenges by exacerbating financial constraints (Kling et al., 2021; Nguyen & Phan, 2020). They may also affect companies’ capital structure by decreasing financial leverage (Ginglinger & Moreau, 2023). In addition, climate risk increases firms’ cash needs by pushing up the cost of external finance. In this sense, Hong et al. (2019) and Huynh et al. (2020) provided evidence that the cost of equity rises with drought conditions. Javadi and Masum (2021) indicated that climate risk raises the cost of bank debts, whereas Huynh and Xia (2021) argued that climate change positively affects the cost of bond loans.

### 2.2 Theoretical background

This article investigates the relationship between climate risk and borrowing discouragement among SMEs. Two opposing views may explain this relationship. The first view suggests that extreme weather events increase a firm's borrowing discouragement. This view is based on the prospect theory that describes how individuals evaluate and make decisions under conditions of uncertainty (Abdellaoui et al., 2007; Edwards, 1996; Tversky & Kahneman, 1992). Climate risk may indeed lead to increased uncertainty and volatility in financial markets (Chenet et al., 2021). This theory offers insights into how climate risk may influence the decision‐making of discouraged borrowers. Given that extreme weather events can negatively impact a firm's operations by damaging assets or causing disruptions (Kleindorfer & Saad, 2005), which the firm perceives as losses, the firm may be averse to the potential losses associated with climate risk. Indeed, climate change poses significant risks to businesses, ranging from supply chain disruptions and increased operational costs to reduced demand for goods and services (Campiglio et al., 2018; Lamperti et al., 2019). Discouraged borrowers, evaluating the potential losses linked to climate risks, may exhibit a reluctance to take on additional debt. The fear of incurring losses from climate‐related damages and increased operating costs could outweigh the potential gains associated with borrowing, which may not sufficiently compensate for the heightened climate risk. This could contribute to a stronger aversion to taking on debt under conditions of significant climate uncertainty. Mbanyele and Muchenje (2022) confirmed that firms confronted with significant uncertainty arising from climate risk become more cautious. Similarly, Huang et al. (2018) showed that firms facing high climate risk in the form of storms, floods, heat waves, and droughts have conservative financial policies that cause them to avoid short‐term leverage.

Extreme weather events can impinge on expected profits and expose firms to more financial constraints (Kling et al., 2021). Physical damage to a firm's assets and supply chain disruption can affect its ability to generate future cash flows (Huang et al., 2022), impacting its capacity to service debt (Capasso et al., 2020). Thus, firms experiencing higher levels of climate risk may be discouraged from taking on additional leverage due to their potential inability to cover the extra expenses linked with increased financial constraints (Ladika & Sautner, 2020; Ni et al., 2022). According to Dafermos et al. (2018), investors and creditors may also perceive climate‐related physical risks as potential threats to a firm's future cash flows and stability. If investors become more risk‐averse due to climate‐related concerns, firms’ capital costs may rise (Noh, 2018). Based on a firm‐level measure of risk, Ginglinger and Moreau (2023) showed that climate risk negatively influences leverage due to the increase in spreads for firms with the greatest risk. Higher financing costs make debt more expensive, potentially increasing the reluctance of companies to borrow.

The second view that may explain the relationship between climate risk and borrowing discouragement among SMEs suggests that climate risk may negatively influence the borrowing discouragement of firms. This view is based on the stakeholder theory, which states that companies should consider the interests of all stakeholders, not just shareholders, in their decision‐making processes (Freeman, 1984). Climate change affects a broad spectrum of stakeholders, including customers, employees, communities, and investors (Cadez et al., 2019). Addressing climate risk demonstrates a commitment to stakeholder well‐being and fosters positive relationships (Kolk & Pinkse, 2007). As stakeholder expectations evolve to include environmental responsibility, companies prioritizing climate risk management enhance their reputations and maintain strong stakeholder relations (Hossain et al., 2023). Therefore, if a company perceives climate risk as an opportunity to invest in resilience and adaptation measures, it might be more inclined to borrow. The funds obtained through borrowing can be used to explore and implement initiatives that enhance the firm's ability to withstand and recover from climate‐related challenges. Thereby, the company can align with changing market demands and regulatory expectations. Governments and regulatory bodies are increasingly focusing on climate‐related disclosure requirements and imposing regulations to mitigate climate change. In 2022, the U.S. Securities and Exchange Commission proposed new rules requiring companies to disclose the risks and impacts of climate change. In 2017, the Economic and Monetary Affairs Committee set similar requirements in Europe. Firms operating in industries with large carbon footprints may face regulatory pressures, affecting their operations and financial performance (Andrews‐Speed, 2016). Furthermore, the pressure of transition and regulatory risks will spur the development of economic and social technology, policies, and laws, which, in turn, will promote the innovation of green technology and green patents (Flammer, 2021). The potential for stricter regulations could motivate firms to use credit to ensure compliance and financial resilience, reducing their reluctance to borrow.

In light of the discussion above, we propose two contrasting hypotheses:

H1a. Climate risk positively influences the discouragement of firms to borrow.
H1b. Climate risk negatively influences the discouragement of firms to borrow.

## 3 DATA, VARIABLES, AND METHODOLOGY

### 3.1 Dependent variable

This study utilizes a substantial sample of SMEs from all countries within the eurozone. The data used in this study come from the Survey of Access to Finance of Enterprises (SAFE), which collects confidential firm‐level information on businesses within the European Union. The survey is conducted by the ECB and the European Commission's Directorate General for Enterprise and Industry. The SAFE database was established in 2009, and since then, it has been updated twice a year. The initial phase of each survey encompasses the second and third quarters of the present year, whereas the succeeding phase encompasses the fourth quarter of the present year and the first quarter of the following year. The microlevel data used in this study span 27 waves, covering the period from the first half of 2009 to the first half of 2022.^1^ The initial database contained almost 260,000 observations of firm–semester pairs, encompassing all European enterprises surveyed. The sample was divided into four categories: company size, economic activity, nation, and size class. In line with Mac an Bhaird et al. (2016) and Anastasiou et al. (2022), this study did not consider entities operating in the financial services, nonprofit, and public administration sectors. Furthermore, observations with missing data in specific categories were excluded from the final dataset. Thus, the dataset used in this study consists of more than 90,000 firm‐level observations.

If a firm requires external funding to accomplish its expansion objectives, SAFE inquiry Q20 seeks clarification about the preferred type of finance that the company seeks to obtain. The responses highlight the necessity of bank credit for businesses as a means of external financing. Furthermore, an analysis of businesses’ responses to question Q7A of the SAFE survey, specifically those responses indicating a decision not to apply for credit due to fear of potential rejection, reveals the hesitancy exhibited by enterprises seeking loans. Thus, in this study, the term “discouraged borrowers” refers to businesses that need credit but refrain from applying for it due to apprehensions about the possibility of being denied. This definition aligns with the perspectives of Anastasiou et al. (2022), Chakravarty and Xiang (2013), Kallandranis and Drakos (2021), Osei‐Tutu and Weill (2023), among others.

In line with the above, our dependent variable ($D_{i}$) is of a dichotomous nature, classifying the *i‐*th firm as discouraged or not, as follows:

(1) $$D_{i}=\left\{\begin{array}{c} \begin{array}{c} 1\;\mathrm{if}\;\mathrm{firm}\;\mathrm{needs}\;\mathrm{credit}\;\mathrm{but}\;\mathrm{did}\;\mathrm{not}\;\mathrm{apply}\;\mathrm{because}\;\mathrm{of}\\ \;\mathrm{fear}\;\mathrm{of}\;\mathrm{rejection}\; \end{array}\\ 0\;otherwise \end{array}\right..$$

**FIGURE 1:**
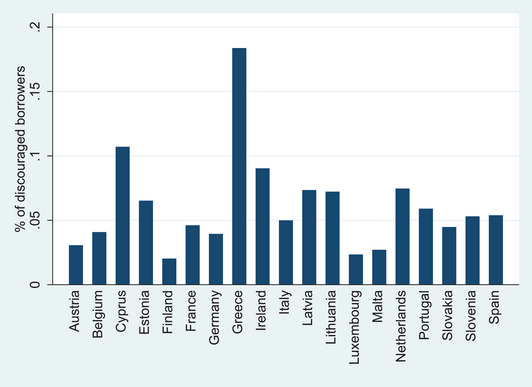
Average percentage of discouraged bank borrowers by country.

**FIGURE 2:**
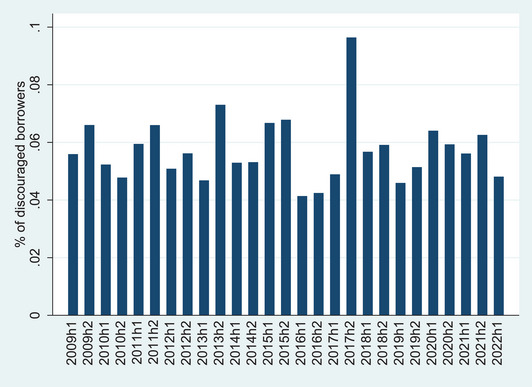
Average percentage of discouraged bank borrowers by time‐wave.

Figures 1 and 2 depict the prevalence of discouraged borrowers by country and by time‐wave, respectively. In Figure 1, which delineates the average percentage of discouraged borrowers across various countries, a discernible pattern emerges, shedding light on the nuanced dynamics. The data indicate notable variations in the prevalence of discouraged borrowers among different nations, with Greece, Ireland, and Cyprus being the countries with higher percentages of discouraged firms and Finland, Luxembourg, and Malta being the countries with lower percentages of discouraged firms. In Figure 2, which portrays the average percentage of discouraged borrowers over time (waves) from 2009H1 to 2022H1, the temporal evolution of potential borrowers’ fear of requesting a bank loan across the entire dataset is unveiled. Throughout the period under examination, we observe both peaks and troughs in discouraged borrower percentages, which could be linked to the broader economic context, regulatory adjustments, and specific events that shaped the financial landscape.

### 3.2 Main explanatory variables—climate risk indices

In order to quantify the effect of climate risk, we employ the climate risk index (CRI) of each country. The CRI created by Germanwatch assesses the measurable consequences of severe weather events, including human fatalities and economic losses. This evaluation is based on data from the Munich Re NatCatSERVICE, which is widely recognized as one of the most dependable and comprehensive databases in this field. CRI serves as a measure of the extent to which countries are exposed to and susceptible to extreme weather events. It is crucial for governments to perceive these indicators as cautionary signals and to implement precautions in anticipation of more frequent and/or intensified catastrophes in the future.

CRI is calculated by Germanwatch as the weighted average ranking of four indicators as follows (model 2):

(2) $$\begin{array}{ccc} \mathrm{CR}I_{i,j} & = & \frac{1}{6}\;\times \mathrm{Rank}\;\mathrm{of}\;\mathrm{Total}\;\mathrm{Number}\;\mathrm{of}\;\mathrm{Death}s_{i,j}\\ & + & \frac{1}{3}\times \mathrm{Rank}\;\mathrm{of}\;\mathrm{Number}\;\mathrm{of}\;\mathrm{Deaths}\;\mathrm{per}\;100,000\;\mathrm{Inhabitant}s_{i,j}\\ & + & \frac{1}{6}\times \mathrm{Rank}\;\mathrm{of}\;\mathrm{Total}\;\mathrm{Losses}\;\mathrm{in}\;\mathrm{US}\;\mathrm{Dollars}\;\mathrm{at}\;\mathrm{PP}P_{i,j}\\ & + & \frac{1}{3}\times Rank\;of\;Losses\;per\;Unit\;of\;GDP_{i,j}, \end{array}$$

where *j* refers to the country and *i* refers to the year. Rank of total number of deaths refers to the total number of human fatalities. Rank of total losses in US dollars at purchasing power parity (PPP) is the total value of losses in US dollars in PPP terms. Both variables are assigned a weight of 1/6 each. Rank of number of deaths per 100,000 inhabitants, which is the number of human fatalities per 100,000 inhabitants, and rank of losses per unit of gross domestic product (GDP), which refers to losses per unit of gross domestic product (GDP), are assigned a weight of 1/3 each. According to Huang et al. (2018), a lower annual CRI provided by Germanwatch indicates greater climate risk. We follow Ni et al. (2022) and scale the index by (−100) to ease the interpretation.

The global CRI indicates a country's level of exposure and vulnerability to extreme weather events. It is calculated as the sum of the exposure index (CRIE) and the vulnerability index (CRIV). CRIE relies on the number of deaths caused by extreme weather events and reflects the extent to which a country is physically susceptible to risks associated with climate change. CRIV is calculated based on the monetary losses incurred as a result of extreme weather events and addresses a country's capacity to respond to climate‐related impacts. These indices are calculated as follows:

(3) $$\begin{array}{ccc} \mathrm{CRI}E_{i,j} & = & \frac{1}{6}\;\times \mathrm{Rank}\;\mathrm{of}\;\mathrm{Total}\;\mathrm{Number}\;\mathrm{of}\;\mathrm{Death}s_{i,j}+\frac{1}{3}\times \mathrm{Rank}\;\mathrm{of}\\ & & \;\mathrm{Number}\;\mathrm{of}\;\mathrm{Deaths}\;\mathrm{per}\;100,000\;\mathrm{Inhabitant}s_{i,j}, \end{array}$$

(4) $$\begin{array}{ccc} \mathrm{CRI}V_{i,j} & = & \frac{1}{6}\;\times \mathrm{Rank}\;\mathrm{of}\;\mathrm{Total}\;\mathrm{Losses}\;\mathrm{in}\;\mathrm{US}\;\mathrm{Dollars}\;\mathrm{at}\;\mathrm{PP}P_{i,j}\\ & + & \frac{1}{3}\times \mathrm{Rank}\;\mathrm{of}\;\mathrm{Losses}\;\mathrm{per}\;\mathrm{Unit}\;\mathrm{of}\;\mathrm{GD}P_{i,j} \end{array}$$

### 3.3 Control variables

Numerous studies have elucidated various aspects that can influence the probability of a particular company becoming a discouraged borrower. In this respect, and in order to reduce the potential for unobserved heterogeneity, we add various empirically common proxies capturing firm‐specific characteristics that influence discouragement. Attributes unique to individual firms have been found to be correlated with a higher probability of experiencing discouragement. In accordance with a priori theoretical considerations, it has been shown that discouragement tends to be more prevalent among smaller and younger firms (Anastasiou et al., 2022; Berger & Udell, 2006; Calabrese et al., 2022; Cole & Sokolyk, 2016; Kallandranis & Drakos, 2021; Mac an Bhaird et al., 2016). In light of this, we include two categorical variables in our regression models for firm size (size) and firm age (*age*), corresponding to specific classes. In addition, four categories denoting the sector in which each firm operates are considered (*activity*). The rationale behind this is to take into account the existence of sectoral heterogeneity, which might be displayed in a firm's decision to apply for a loan (Cole & Sokolyk, 2016; Freel et al., 2012; Mac an Bhaird et al., 2016). The idea is that including these measures will eradicate disparities in financial access that are unique to each sector and that result in the sector impacting a firm's financing goals and results. Finally, the type of ownership is included as a possible determinant of borrower discouragement to capture the potential effect of firms’ financial conditions (Anastasiou et al., 2023; Mac an Bhaird et al., 2016). Although we expect that the type of ownership will affect the borrowing decision, the specific impact is not easily predictable ex ante (Freel et al., 2012).

We also include four dummy variables compared to the previous wave that indicate the company's profitability (profit) (in order to control for the finance seeking and worthiness of the SME); level of interest expenses (interest expenses) (given that companies tend to be deterred by elevated interest‐rate expenditures); financial leverage (leverage) (as firms experiencing strong financial pressure are likely to be discouraged); and credit history (credit history) (since a firm with a deteriorating credit outlook is more likely to have a weakening credit rating and thus stricter criteria for obtaining bank loans, leading to probable discouragement) (Bottazzi et al., 2014; Cowling et al., 2016; Ferrando & Mulier, 2022; Ferrando et al., 2017; Romano et al., 2001).

The macro‐financial environment in which businesses operate also affects their likelihood of applying for bank loans. In order to account for the potential influence of country‐specific characteristics and financial market conditions on firms’ responses, we enhance our regression model by also incorporating pertinent country‐specific and financial market indicators. This allows us to investigate whether the likelihood of firm discouragement can be attributed to variations across different countries. For this reason, we incorporate the real GDP growth rate, unemployment rate, HICP inflation rate, and the 10‐year government bond yield by country into our regression models (Anastasiou et al., 2022; Chakravarty & Xiang, 2013; Mac an Bhaird et al., 2016; Moro et al., 2020).

Table 1 defines all the variables used in our analysis, wh ereas Table 2 reports the main descriptive statistics for each variable.

**TABLE 1:** Definition of variables (Baseline models).

Variable	Definition	Expected sign
**Dependent variable**		
Discouraged	1, if a company needs external financing but did not apply because of possible rejection; 0, otherwise	n/a
**Climate risk variables**		
CRI	Climate risk index as measured by GermanWatch	**+/−**
CRIV	The climate risk vulnerability subcomponent	**+/−**
CRIE	The climate risk exposure subcomponent	**+/−**
**Firm specific‐controls**		
Age	Categorical variable equal to 1 if the firm is 10 years or more; 2 if the firm is between 5 and 10 years old; 3 if the firm is between 2 and 5 years old; 4 if the firm is less than 2 years old	+
Size	Categorical variable equal to 1 for micro firms (1–9 employees); 2 for small firms (10–49 employees); 3 for medium‐sized firms (50–249 employees)	–
Ownership	Categorical variable equal to 1 if the firm is family‐owned; 2 if business associates; 3 if venture capital firms or business angels; 4 if natural person‐one owner only; 5 if public shareholders	+/−
Activity	Categorical variable equal to 1 if the firm belongs to the construction sector; 2 if the firm belongs to the wholesale or retail trade sector; 3 if the firm belongs to the services sector; 4 if the firm belongs to the transport sector	+/−
Profit	Dummy variable equal to 1*–*6 months; 0 otherwise	+
Interest expenses	Dummy variable equal to 1 if the firm's interest expenses decreased in the past 6 months; 0 otherwise	–
Leverage	Dummy variable equal to 1 if the firm's leverage decreased in the past 6 months; 0 otherwise	–
Credit history	Dummy variable equal to 1 if the firm's credit history deteriorated in the past 6 months; 0 otherwise	+
**Country specific‐controls**		
RGDP	Real GDP annual growth	–
Yield	10‐year government bond yield	+
Unempl	Unemployment rate	+
Inflation	HICP inflation rate	+/−

Abbreviation: GDP, gross domestic product.

**TABLE 2:** Summary statistics.

Variable	Obs	Mean	Std. Dev.	Min	Max
Discouraged	178,841	0.06	0.23	0	1
CRI	184,953	63.43	21.00	17.33	126.17
CRIV	184,808	35.76	11.02	1.66	57.00
CRIE	184,953	27.75	14.27	5.33	70.16
Age	258,927	1.23	0.59	1	4
Size	261,879	2.05	0.98	1	4
Ownership	243,645	3.14	1.40	1	5
Activity	239,757	2.82	1.18	1	4
Profit	255,661	2.11	0.81	1	2
Interest expenses	223,115	1.93	0.64	1	2
Leverage	166,276	2.06	0.65	1	2
Credit history	242,188	1.86	0.57	1	2
RGDP	261,879	1.18	4.16	−13.3	23.2
Yield	261,879	2.03	2.60	−0.57	25.07
Unempl	261,879	10.05	5.52	3.00	28.00
Inflation	255,788	0.85	1.24	−2.30	10.66

Abbreviation: CRI, climate risk index.

### 3.4 Econometric methodology

In order to model the impact of climate risk on discouragement, we employ a pooled panel probit model with robust standard errors that reads as follows:

(5) $$\begin{array}{ccc} \mathrm{Prob}(D_{i,j,t}=1|N=1) & = & \lambda_{0}+\lambda_{\xi }\cdot {\mathrm{climate}}_{j,t}\\ & & +\;\sum_{k=1}^{N}\theta_{k}\cdot {\mathrm{Controls}}_{i,j,t}\\ & & +\;\tau_{t}+\varepsilon_{i,j,t},\xi =1,2,3, \end{array}$$

where i,j,t denote firm, country and time (semester/wave). Thus, consistent with the definition of discouragement described above, we model the probability of a firm being discouraged ($D_{i}$), given that this firm needs a bank loan (N=1). The variable $\mathrm{climat}e_{j,t}$ denotes each proxy for climate risk (namely, CRI, CRIE, and CRIV) in country *j* and period *t*. Controls is a vector of contemporaneous firm‐level and macro control variables as described above. Wave‐time dummies are also included in all models (denoted by *τ_t_*) to absorb the potential unobserved time‐varying effects (Cowling & Sclip, 2022).

All regressions are estimated with a probit model with heteroskedasticity‐ and autocorrelation‐consistent asymptotic standard errors, computed according to Newey and West (1987). We use the maximum likelihood estimator to estimate the above probit models, which is mathematically formulated as follows:

(6) $$\ln L=\sum_{j\in S}^{w_{j}}\;\ln\Phi \left(x_{j}\beta \right)+\sum_{j\notin S}^{w_{j}}\;\ln\left\{1-\Phi (x_{j}\beta )\right\}$$

where *Φ* is the cumulative normal and *w_j_* denotes the optional weights.

It is important to understand that the calculated coefficients cannot be used to assess the relative magnitude of the variables. Marginal effects are necessary for the purpose of comparing various factors. The marginal effect computed describes the relationship between the probability of discouragement and a change of one percentage point in a continuous explanatory independent variable.

## 4 EMPIRICAL FINDINGS

### 4.1 Baseline results

Table 3 presents the marginal effects for three different probit model estimations explaining the binary variable discouragement. Each column represents the three alternative measures of climate risk, namely CRI, CRIE, and CRIV.

**TABLE 3:** Baseline probit results.

	(1)	(2)	(3)
VARIABLES	Pr(discouraged borrower = 1)		
**CRI**	**0.003** ^***^		
	**[0.000]**		
**CRIV**		**0.004** ^***^	
		**[0.001]**	
**CRIE**			**0.004** ^***^
			**[0.001]**
Age	0.003^**^	0.003^**^	0.003^**^
	[0.002]	[0.002]	[0.002]
Size	−0.019^***^	−0.019^***^	−0.019^***^
	[0.001]	[0.001]	[0.001]
Family or entrepreneurs	−0.007	−0.007	−0.008
	[0.009]	[0.009]	[0.009]
Other firms or business associates	−0.009	−0.009	−0.010
	[0.009]	[0.009]	[0.009]
Venture capital firms or business angels	0.025	0.026	0.025
	[0.017]	[0.017]	[0.017]
A natural person, one owner only	−0.002	−0.002	−0.002
	[0.009]	[0.009]	[0.009]
Construction	0.006	0.006	0.006
	[0.004]	[0.004]	[0.004]
Trade	−0.012^***^	−0.011^***^	−0.012^***^
	[0.003]	[0.003]	[0.003]
Services	−0.008^***^	−0.007^***^	−0.007^***^
	[0.003]	[0.003]	[0.003]
Profit	0.014^***^	0.014^***^	0.013^***^
	[0.001]	[0.001]	[0.001]
Interest expenses	−0.016^***^	−0.016^***^	−0.015^***^
	[0.002]	[0.002]	[0.002]
Leverage	−0.004^***^	−0.004^***^	−0.004^***^
	[0.001]	[0.001]	[0.001]
Credit history	0.024^***^	0.024^***^	0.024^***^
	[0.002]	[0.002]	[0.002]
RGDP	−0.002^***^	−0.002^***^	−0.002^***^
	[0.000]	[0.000]	[0.000]
Yield	0.014^***^	0.015^***^	0.014^***^
	[0.001]	[0.001]	[0.001]
Unempl	0.001^***^	0.001^***^	0.001^***^
	[0.000]	[0.000]	[0.000]
Inflation	−0.001	−0.002	−0.002
	[0.003]	[0.003]	[0.003]
Time (wave) dummies	Included	Included	Included
Observations	54,722	54,695	54,722
Wald *χ* ^2^	1565.42	1502.25	1592.98
Log pseudolikelihood	−12032.41	−12009.62	−12021.38
Pseudo *R* ^2^	0.075	0.073	0.076

*Note*: (a) Sample period: semiannual data from 2009 to 2022, (b) heteroskedasticity‐ and autocorrelation‐consistent (HAC) asymptotic standard errors reported in parentheses are computed according to Newey and West (1987), and (c) significance levels are indicated as follows: ∗*p* < 0.10; ∗∗*p* < 0.05; and ∗∗∗*p* < 0.01. Bold values denote the key variables of interest.

Abbreviation: CRI, climate risk index.

With respect to the parameters of interest (climate risk indices), the data suggest a number of interesting observations. Notably, in all columns, the marginal effects of the three indices are positive and statistically significant at the 1% level, suggesting that an increase in climate risk (unrelated to fundamentals) increases the number of discouraged firms. Intuitively, with a 1% increase in CRI, we find an increase of 0.3% in the likelihood of observing a discouraged borrower, whereas for CRIV and CRIE, this effect is increased by 0.4% for both indices. These results are not only statistically but also economically significant. They confirm that climate risk exhibits a positive relationship with the discouragement of borrowers, supporting our hypothesis H1a and the previously reported mechanisms of the prospect theory (Abdellaoui et al., 2007; Edwards, 1996). As firms face substantial risks from climate change, this could lead to a strong aversion to taking on debt under conditions of heightened climate risk. Therefore, SMEs become less likely to ask for a loan as climate change risk increases. The key finding is that negative economic shocks resulting from extreme weather events can increase general uncertainty, which intensifies information asymmetries between borrowers and lenders (Mishkin, 2011). Within such an uncertain environment, many failed loan applications may discourage borrowers (Cowling et al., 2016).

Regarding the marginal effects of the control variables, we observe that they carry the same sign and level of significance across all facets and coincide with the theoretical predictions and previous empirical research. For instance, if a company's credit history, a variable that is indirectly linked to information opacity, and profitability prospects have deteriorated over the preceding 6 months, this leads to intensified discouragement. Indeed, a firm with a poor credit history is considered riskier by banks and is more likely to be discouraged (Anastasiou et al., 2022, 2023; Cowling et al., 2016; Ferrando & Mulier, 2022). Variables associated with companies’ economic and financial situation over the preceding 6 months are highly relevant to the likelihood of discouragement. A decrease in the total amount of interest expenses that companies have incurred and a decline in the debt‐to‐assets ratio are associated with a reduced likelihood of feeling discouraged from applying for a bank loan (Anastasiou et al., 2022, 2023; Ferrando & Mulier, 2022; Mac an Bhaird et al., 2016; Xiang et al., 2015). Moreover, the structural features of a company have a big impact on whether it decides to apply for a loan. Unsurprisingly, we find that smaller firms exhibit higher levels of discouragement on average, which is in line with previous evidence in the literature (Anastasiou et al., 2023; Freel et al., 2012; Kallandranis et al., 2023), whereas for age, a positive impact at 5% significance level is reported. Hence, younger firms are more likely be discourage borrowers supporting Han et al. (2009), Calabrese et al. (2022), and Brown et al. (2022). Regarding industry effects, with transport as a reference group, SMEs operating in the trade and services sectors are less likely to be discouraged, contrary to firms in construction, which enter the estimation with no significant effect. Finally, the macroeconomic variables were also found to have signs compatible with the prior literature. In particular, within an intensifying macroeconomic environment, the probability for firms to be discouraged is diminished confirming the finding of Mac an Bhaird et al. (2016). Higher government yields and unemployment rate are both associated with heightened discouragement, whereas inflation exerts no impact similarly to Mac an Bhaird et al. (2016) and Moro et al. (2020).

Overall, our findings offer useful insights into the assessment of discouragement among European SMEs. They enhance our understanding of the elements that influence borrowing discouragement and can reveal ways for managing climate risk in credit markets.

### 4.2 Robustness checks

In this section, we provide a set of robustness checks in order to ensure the validity of the baseline results.

#### 4.2.1 Heckman correction

When a nonrandom subset is chosen from the entire population, standard regression analysis produces the well‐known sample selection bias. In order to address a potential bias in selection stemming from our observation that only businesses have credit requirements, we employ a probit model with a Heckman (1981) adjustment. This selection model consists of two equations. The initial equation is known as the selection model, which employs a probit model to predict whether a firm needs a bank loan. The second equation represents the outcome model, which determines whether a firm seeking a bank loan is discouraged. It should be mentioned, though, that because the waves of the SAFE survey contain different cross‐sections, the sample is actually a pseudo‐panel by design. A possible criticism of Heckman's (1981) initial study is that it was founded on panel datasets, which renders such an undertaking impracticable in our particular context. However, we adopted this estimate as a robustness test in light of prior research in the field (see, for instance, Anastasiou et al., 2022, 2023; Brown et al., 2022).

Our general setup consists of the selection equation that models the probability of a firm needing a loan. It can be mathematically formulated as follows:

(7) $$\begin{array}{ccc} \mathrm{Prob}\;(N_{i,j,t}=1) & = & \lambda_{0}+\lambda_{1}\cdot {\mathrm{competition}}_{j,t-1}+\sum_{k=1}^{N}\theta_{k}\cdot {\mathrm{Controls}}_{i,j,t}\\ & & +\;\tau_{t}+\varepsilon_{i,j,t}. \end{array}$$

Then, we model the so‐called outcome equation as follows:

(8) $$\begin{array}{ccc} \mathrm{Prob}\;(D_{i,j,t}=1|N=1) & = & \lambda_{0}+\lambda_{\xi }\cdot {\mathrm{climate}}_{j,t}+\sum_{k=1}^{N}\theta_{k}\cdot {\mathrm{Controls}}_{i,j,t}\\ & & +\;\psi \cdot \left(\rho \cdot \sigma_{\varepsilon }\right)+\tau_{t}+u_{i,j,t},\xi =1,2,3, \end{array}$$

where i,j,t denote firm, country, and time (semester/wave). Consistent with the definition of discouragement described above, we model the probability of a firm being discouraged ($D_{i}$), given that this firm needs a bank loan (N=1). The variable $\mathrm{climat}e_{j,t}$ denotes each proxy for climate risk (namely, CRI, CRIE, and CRIV) in country *j* and period *t*.

We assume that $\left(\varepsilon_{i,j,t},u_{i,j,t}\right)$ follows a bivariate normal distribution with

(9) $$\left(\begin{array}{c} \varepsilon_{i,j,t}\\ u_{i,j,t} \end{array}\right)∼N\left[\left(\begin{array}{c} 0\\ 0 \end{array}\right),\left(\begin{array}{cc} \sigma_{\varepsilon }^{2} & \rho \sigma_{\varepsilon }\\ \rho \sigma_{\varepsilon } & \sigma_{u}^{2} \end{array}\right)\right],$$

where (ρ) is the correlation between $(\varepsilon_{i,j,t},u_{i,j,t}),$ whereas ψ is the inverse Mill's ratio denoting the non‐selection hazard.

As per the methodology of Bremus and Neugebauer (2018), Ćehajić and Košak (2022), and Ferrando et al. (2017), competition is included as a selection variable. A variable in the SAFE database is given a value of 1 if the company considers competition to be its top concern or priority and 0 otherwise. Companies that are confronted with fierce market competition, which leads to a decrease in both profits and sales, might have a greater need for credit but exhibit a diminished propensity to seek out bank loans. We anticipate that a bank's determination to grant a loan will not be impacted by a firm's discouragement. This prediction satisfies the exclusion limitation criterion because it implies that the influence of discouragement is limited to the demand for rather than the supply of credit. The marginal effects of Heckman probit models incorporating sample selection are presented in Table 4. Our primary discoveries continue to endure.

**TABLE 4:** Further analysis: Heckman correction.

	(1)	(2)	(3)
VARIABLES	Pr(discouraged borrower = 1)		
**CRI**	**0.011** ^***^		
	**[0.002]**		
**CRIV**		**0.016** ^***^	
		**[0.004]**	
**CRIE**			**0.012** ^***^
			**[0.003]**
Age	0.008^*^	0.008^*^	0.008^*^
	[0.005]	[0.005]	[0.004]
Size	−0.076^***^	−0.076^***^	−0.069^***^
	[0.018]	[0.017]	[0.014]
Family or entrepreneurs	−0.041	−0.051^*^	−0.045
	[0.029]	[0.029]	[0.028]
Other firms or business associates	−0.046	−0.059^**^	−0.050^*^
	[0.029]	[0.030]	[0.028]
Venture capital firms or business angels	0.043	0.035	0.039
	[0.044]	[0.045]	[0.044]
A natural person, one owner only	−0.036	−0.049^*^	−0.038
	[0.027]	[0.029]	[0.027]
Construction	0.022^**^	0.023^**^	0.025^**^
	[0.011]	[0.010]	[0.010]
Trade	−0.012^*^	−0.008	−0.011
	[0.007]	[0.007]	[0.006]
Services	0.005	0.006	0.007
	[0.008]	[0.008]	[0.008]
Profit	0.033^***^	0.034^***^	0.031^***^
	[0.006]	[0.006]	[0.006]
Interest_expenses	−0.019^***^	−0.019^***^	−0.018^***^
	[0.004]	[0.004]	[0.004]
Leverage	−0.020^**^	−0.021^**^	−0.017^**^
	[0.010]	[0.010]	[0.008]
Credit_history	0.058^***^	0.059^***^	0.054^***^
	[0.011]	[0.011]	[0.009]
RGDP	−0.007^***^	−0.007^***^	−0.007^***^
	[0.002]	[0.002]	[0.002]
Yield	0.036^***^	0.038^***^	0.034^***^
	[0.007]	[0.007]	[0.006]
Unempl	0.004^***^	0.004^***^	0.004^***^
	[0.002]	[0.002]	[0.001]
Inflation	−0.003	−0.007	−0.005
	[0.007]	[0.007]	[0.007]
Time (wave) dummies	Included	Included	Included
Wald *χ* ^2^	1659.51^***^	1576.07^***^	1652.08^***^
Log pseudolikelihood	45615.34	−45767.73	−45624.66
Observations	54,722	54,695	54,722

*Note*: (a) Sample period: semiannual data from 2009 to 2022, (b) heteroskedasticity‐ and autocorrelation‐consistent (HAC) asymptotic standard errors reported in parentheses are computed according to Newey and West (1987), and (c) significance levels are indicated as follows: ∗*p* < 0.10; ∗∗*p* < 0.05; and ∗∗∗*p* < 0.01. Bold values denote the key variables of interest.

Abbreviation: CRI, climate risk index.

#### 4.2.2 Treating endogeneity concerns

The findings above may be influenced by endogeneity issues. It is widely recognized that missing variables are a frequent cause of endogeneity. In the previously described models, we incorporate several firm and macro features to address the presence of unobserved differences. Nevertheless, there may be other latent factors that influence both the climate risk variables and discouraged borrowers yet have not been incorporated into the model. Undoubtedly, intangible elements such as shifts in the regulatory landscape, the corporate ethos, and the risk appetite of the CEO can influence a firm's inclination to refrain from seeking a bank loan. Endogeneity can also arise via reverse causality. In this case, it is likely that climate risk contributes to discouragement, but this correlation could also operate in the other direction. Climate risk commonly encompasses environmental adversities such as severe weather phenomena, rising sea levels, and temperature fluctuations. These adversities are influenced by intricate and extensive physical processes associated with the Earth's atmosphere, seas, and ecosystems. They remain unaffected by the emotional or financial conditions of individuals or particular groups, such as debtors who are feeling disheartened. Thus, the presence of reverse causality does not align with our study's findings.

In order to address the issue of endogeneity, we utilize an instrumental variable (IV) approach known as IV‐probit, as suggested by Moscalu et al. (2020) and Popov and Udell (2012). This method requires the identification of valid instruments that satisfy two conditions: (i) the instruments must exhibit a strong correlation with the main explanatory variable, which is the endogenous regressor (climate risk) and (ii) the instruments must be exogenous, meaning that they cannot be correlated with the error term. In order to achieve this objective, we recalibrate our initial specification using an IV‐probit model. The instruments we utilize are as follows:

- Lagged values of climate risk variables. The idea is that the lagged values of the climate risk variables are correlated with the contemporaneous values but are not affected by the current value of the error term. Using lagged variables as instruments for contemporaneous (climate risk) variables is a common approach in regression types such as IV and generalized method of moments (see, among others, Laeven et al., 2015). Incorporating lagged variables can attenuate contemporaneous interdependencies and potential biases; however, it is imperative to acknowledge that they might not entirely alleviate endogeneity concerns akin to those addressed by IVs. Climate‐related risks, encompassing fluctuations in temperature, alterations in sea levels, and shifts in climate policies, exert pervasive repercussions transcending the immediate temporal domain. These ramifications extend beyond present instances of discouraged borrowing, potentially influencing forthcoming occurrences as well.
- As a second instrument, we employ the population density following Huang et al. (2018). Population density should be correlated with climate risk but not directly related to discouraged borrowers. For example, areas with higher population density might be more vulnerable to certain climate risks or might have different adaptive capacities.

In order to assess the soundness of our approach and the validity of our instrumentation strategy, we conduct two diagnostic tests. Initially, we examine the strength of our instruments by assessing their correlation with the endogenous variable. In order to do this, we rely on the first stage *F*‐statistic of the instruments that have been omitted (*F*‐1st (excluding IV)). It is evident from the results shown in Table 5 that *F*‐1st (excluding IV) is in each specification significantly greater than the threshold level of 10 (Stock & Yogo, 2005). This suggests that the instruments utilized are valid and not “weak,” and that there is a correlation between the instruments and the predictor.

**TABLE 5:** Endogeneity concerns—instrumental variable (IV)‐probit results.

	(1)	(2)	(3)
VARIABLES	Pr(discouraged borrower = 1)		
**CRI**	**0.051** ^***^		
	**[0.004]**		
**CRIV**		**0.049** ^***^	
		**[0.009]**	
**CRIE**			**0.080** ^***^
			**[0.006]**
Age	0.016	0.015	0.017
	[0.015]	[0.015]	[0.015]
Size	−0.216^***^	−0.214^***^	−0.211^***^
	[0.012]	[0.012]	[0.012]
Family or entrepreneurs	−0.073	−0.087	−0.079
	[0.079]	[0.079]	[0.079]
Other firms or business associates	−0.143^*^	−0.158^*^	−0.147^*^
	[0.083]	[0.083]	[0.083]
Venture capital firms or business angels	0.131	0.129	0.132
	[0.124]	[0.124]	[0.124]
A natural person, one owner only	−0.097	−0.118	−0.094
	[0.080]	[0.079]	[0.080]
Construction	0.037	0.038	0.044
	[0.031]	[0.031]	[0.031]
Trade	−0.078^***^	−0.065^**^	−0.074^***^
	[0.025]	[0.026]	[0.025]
Services	−0.077^***^	−0.074^***^	−0.073^***^
	[0.024]	[0.024]	[0.024]
Profit	0.164^***^	0.165^***^	0.160^***^
	[0.012]	[0.012]	[0.012]
Interest expenses	−0.175^***^	−0.177^***^	−0.172^***^
	[0.015]	[0.015]	[0.015]
Leverage	−0.045^***^	−0.040^***^	−0.044^***^
	[0.014]	[0.014]	[0.014]
Credit history	0.242^***^	0.241^***^	0.240^***^
	[0.016]	[0.016]	[0.016]
RGDP	−0.003^***^	−0.003^***^	−0.003^***^
	[0.000]	[0.000]	[0.000]
Yield	0.005^***^	0.005^***^	0.005^***^
	[0.001]	[0.001]	[0.001]
Unempl	0.003^***^	0.003^***^	0.003^***^
	[0.001]	[0.001]	[0.001]
Inflation	−0.001	−0.002	−0.002
	[0.003]	[0.003]	[0.003]
Constant	−1.556^***^	−1.430^***^	−1.453^***^
	[0.116]	[0.117]	[0.114]
Time (wave) dummies	Included	Included	Included
Observations	54,713	54,686	54,713
Wald *χ* ^2^	1564.44	1500.60	1593.38
Log pseudolikelihood	41177.58	70326.642	60933.818
*F*‐1st (excl. IV)	23.70	57.70	38.98

*Note*: (a) Sample period: semiannual data from 2009 to 2022, (b) heteroskedasticity‐ and autocorrelation‐consistent (HAC) asymptotic standard errors reported in parentheses are computed according to Newey and West (1987), and (c) significance levels are indicated as follows: ∗*p* < 0.10; ∗∗*p* < 0.05; and ∗∗∗*p* < 0.01. Bold values denote the key variables of interest.

Abbreviation: CRI, climate risk index.

Furthermore, our IV technique is considered valid if the instruments adhere to the exclusion limitation. In other words, the error term in the second stage should be independent of the eliminated instruments. In order to evaluate this hypothesis, we conduct an over‐identification test. We find that the null hypothesis is not rejected; hence, the instruments are independent (orthogonal) from (to) the error term and satisfy the exclusion requirement. Overall, the results remain unaffected.

#### 4.2.3 Alternative definition of discouragement

Thus far, our definition of discouraged borrowers has encompassed companies that require financing but choose not to apply for it due to fear of rejection. Indeed, when non‐creditworthy enterprises choose not to seek loans, this is not an issue because it indicates that discouragement serves as an effective self‐regulating mechanism. Consistent with this perspective, we adhere to the stringent definition of discouraged borrowers proposed by Kon and Storey (2003). This definition only includes SMEs that are creditworthy and have the potential to obtain loans but choose not to apply due to their fear of being denied.

As a result, we limit our sample to companies with good credit ratings. In order to determine the creditworthiness of enterprises, we adhere to the methodology established by Petersen and Rajan (1994), which entails exclusively considering firms that currently possess an active bank loan, line of credit, or overdraft facility. The rationale is that companies with continuous access to bank credit are more likely to be deemed creditworthy, as they have already proven their capability to repay their loans. To that end, we reestimate our models for a subset of companies that currently possess active bank credit and confirmed in the SAFE survey that their credit quality had increased.

Table 6 shows the marginal effects when we employ this alternative definition of discouragement, namely, when we take into account the “good borrowers.” The results confirm our earlier findings that climate risk significantly increases firms’ likelihood of being discouraged.

**TABLE 6:** Alternative definition for discouragement.

	(1)	(2)	(3)
VARIABLES	Pr(discouraged borrower = 1)		
**CRI**	**0.002** ^***^		
	**[0.001]**		
**CRIV**		**0.004** ^***^	
		**[0.001]**	
**CRIE**			**0.005** ^***^
			**[0.001]**
Age	−0.001	−0.000	−0.001
	[0.002]	[0.002]	[0.002]
Size	−0.009^***^	−0.009^***^	−0.009^***^
	[0.001]	[0.001]	[0.001]
Family or entrepreneurs	−0.003	−0.003	−0.003
	[0.009]	[0.009]	[0.009]
Other firms or business associates	−0.015	−0.015	−0.015
	[0.009]	[0.009]	[0.009]
Venture capital firms or business angels	0.003	0.004	0.003
	[0.016]	[0.016]	[0.016]
A natural person, one owner only	−0.003	−0.005	−0.003
	[0.009]	[0.009]	[0.009]
Construction	0.001	0.002	0.002
	[0.004]	[0.004]	[0.004]
Trade	−0.006^*^	−0.006^*^	−0.005^*^
	[0.003]	[0.003]	[0.003]
Services	−0.003	−0.004	−0.003
	[0.003]	[0.003]	[0.003]
Profit	0.010^***^	0.010^***^	0.010^***^
	[0.001]	[0.001]	[0.001]
Interest expenses	−0.012^***^	−0.012^***^	−0.012^***^
	[0.002]	[0.002]	[0.002]
Leverage	0.003^**^	0.003^**^	0.003^**^
	[0.002]	[0.002]	[0.002]
Credit history	Omitted	Omitted	Omitted
RGDP	−0.001^**^	−0.001^**^	−0.001^***^
	[0.000]	[0.000]	[0.000]
Yield	0.008^***^	0.008^***^	0.008^***^
	[0.001]	[0.001]	[0.001]
Unempl	0.000	0.000	0.001^**^
	[0.000]	[0.000]	[0.000]
Inflation	−0.004	−0.004	−0.004
	[0.003]	[0.003]	[0.003]
Time (wave) dummies	Included	Included	Included
Observations	22,468	22,462	22,468
Wald *χ* ^2^	272.45	249.73	277.78
Log pseudolikelihood	−2980.30	−2970.73	−2978.60
Pseudo *R* ^2^	0.046	0.042	0.046

*Note*: (a) Sample period: semiannual data from 2009 to 2022, (b) heteroskedasticity‐ and autocorrelation‐consistent (HAC) asymptotic standard errors reported in parentheses are computed according to Newey and West (1987), and (c) significance levels are indicated as follows: ∗*p* < .10; ∗∗*p* < 0.05; and ∗∗∗*p* < 0.01.

Abbreviation: CRI, climate risk index.

### 4.3 Mapping the impact of climate risk on discouragement across different sample sub‐groups

We examine and measure the variations in self‐rationing (discouragement) patterns by analyzing certain subsets of firms that showcase distinct combinations of attributes. Specifically, we compute the probability of discouragement under various scenarios based on a single characteristic, such as size, age, and credit history, and given that a country experiences intensified climate risk.

With the aim to achieve this, we restrict our sample for climate risk (CRI) being above its median, and we also limit our variable selection to only those that may clearly act as credit rationing predictors identifying the adverse selection problem. Indeed, size, age, and credit history serve as qualitative proxy variables addressing risk characteristics on both the credit supply and demand side and capturing aspects of information flows and transparency that can affect the cost of capital. In the literature, this is considered a standardized approach to analyzing firms’ lending behavior (Kallandranis et al., 2023). Under this premise, we examine the potential effect of the underlying variables, showing that the self‐rationing problem is evident for firms with certain characteristics and confirming all previous studies. In this context, we also explore how climate risk affects firms’ financing choices and contributes to the financial exclusion of SMEs, going beyond the traditional rationing problem.

The relevant results for the mean predicted probabilities are reported in Table 7. We first compare firms across size classes. Size exerts a monotonic downward drop (from micro to medium‐sized firms). The probability of a micro firm being discouraged is 9.4%, whereas for a medium‐sized firm it is 3.4%. This produces a predicted probability ratio (PPR) of 2.76, suggesting that micro firms are 2.76 times more likely to be discouraged than their larger counterparts. When climate risk is introduced, the effect is fuelled across all size classes. This produces a PPR above unity, which ranges from 1.10 to 1.14 across size classes, implying that discouragement is enhanced significantly when climate risk is considered. When considering the age effect, it is evident that rather young SMEs are 1.25 times more likely to be discouraged than mature ones. When climate risk is included in the analysis, the PPRs are all higher than unity across all facets of outcomes, indicating that the climate effect magnifies the results. The credit history trait follows the same pattern, confirming that when a firm's credit outlook is poor, the likelihood of the firm being discouraged is more than four times higher than those firms with a positive credit outlook. A similar picture emerges when climate risk is included, with the PPR ranging between 1.12 and 1.15, implying that the probability of being discouraged is higher for SMEs experiencing high climate risk.

**TABLE 7:** Discouraged borrowers mapped.

Variable/Sample sub‐group	Without climate risk impact (%)	With climate risk impact (%)
**Size**		
Micro: 1 < Size < 9 employees	9.4	10.4
Small: 10 < Size < 49 employees	5.7	6.5
Medium: 50 < Size < 249 employees	3.4	3.9
**Age**		
Age < 2 years	7.7	8.6
2 years < Age < 5 years	7.4	8.2
5 years < Age < 10 years	7.3	8.2
Age > 10 years	6.2	7.0
**Credit history**		
Improved	3.3	3.8
Deteriorated	13.6	15.3

*Note*: This table presents the predicted probabilities for three distinct traits (sub‐groups), namely, firm age, size, and credit history (quality) for the baseline probit results with CRI.

## 5 CONCLUSIONS AND POLICY IMPLICATIONS

The research presented in this article underscores the multifaceted implications of climate risk for discouraged borrowers among European SMEs. The empirical evidence reveals that climate risk is a significant factor influencing SMEs’ decisions to self‐ration credit, thereby contributing to the prevailing phenomenon of discouraged borrowing.

Using a confidential dataset from the ECB for all eurozone countries, we find that climate risk has a positive effect on discouraged borrowers, supporting the prospect theory perspective. This result suggests that negative economic shocks as a result of extreme weather events can generate an increase in general uncertainty that intensifies information asymmetries between borrowers and lenders. Thus, within an uncertain climate environment, a possibly high number of failed loan applications leads to discouraged borrowers. Our results are robust to a set of analyses including additional control variables and Heckman correction and persist after endogeneity concerns are addressed.

Several key insights and policy implications emerge from our investigation, offering guidance for policymakers, financial institutions, and stakeholders concerned with fostering economic resilience in the face of climate change. First, our study adds a nuanced layer to the understanding of the relationship between climate risk and credit markets by emphasizing the demand‐side dynamics. Traditionally, the literature has predominantly focused on supply‐side issues, neglecting the role of borrower perceptions and responses to emerging risks. Recognizing the significance of climate risk in shaping credit‐related decisions, policymakers, and financial institutions should pivot toward a more comprehensive approach that addresses both supply and demand dynamics when crafting effective strategies. Furthermore, our findings emphasize the need for proactive measures to protect economies from climate shocks. Policymakers and central banks should consider integrating climate risk assessments into their regulatory frameworks and encouraging financial institutions to incorporate climate risk considerations in their lending practices. This could involve the development of standardized metrics for assessing climate risk exposure, facilitating informed decision‐making and risk management strategies.

This research underscores the urgent need for a paradigm shift in addressing climate risk within the context of credit markets. By acknowledging the impact of climate risk on discouraged borrowers, central banks and financial institutions can work collaboratively to foster a resilient and adaptive economic environment. This study contributes to the evolving discourse on climate change adaptation, providing insights that can inform policy decisions aimed at promoting sustainable economic growth and development in the face of environmental challenges. In terms of direction of future research, future studies could adopt a longitudinal instead of a pooled approach to examine how the relationship between climate risk and SME borrowing behavior evolves over time and across different economic cycles. Moreover, investigating how different sectors within the SME category respond to climate risk could yield more targeted insights and policy recommendations. Finally, examining the impact of CEOs’ climate risk perceptions on the likelihood of being discouraged would also be interesting.

## Footnotes

1 Given the availability of the Germanwatch index up to 2019, our final sample is restricted from 2009 to 2019. Consequent, our analysis will be based on 19 waves.
